# Efficacy of injection of autologous adipose tissue in the treatment of patients with complex and recurrent fistula-in-ano of cryptoglandular origin

**DOI:** 10.1007/s10151-024-02963-x

**Published:** 2024-07-09

**Authors:** S. Guillaumes, N. J. Hidalgo, I. Bachero, R. Pena, S. T. Nogueira, J. Ardid, M. Pera

**Affiliations:** Hospital Clinic de Barcelona, Barcelona, Spain

**Keywords:** Anal fistula, Recurrent, Adult stem cells, Adipose tissue, Regenerative medicine

## Abstract

**Background:**

Adipose tissue injections, a rich source of mesenchymal stem cells, have been successfully used to promote anal fistula healing. This study aimed to investigate the efficacy of adipose tissue injection in treating patients with complex and recurrent fistulas of cryptoglandular origin.

**Methods:**

We conducted a prospective, single-center, open-label, non-randomized, interventional clinical trial from January 2020 to December 2022. We enrolled nine patients, who were evaluated after at least 12 months of follow-up. All patients had seton removal, fistula tract excision or curettage, and a mucosal flap if possible or, alternatively, an internal opening suture. We used a commercially available system to collect and process adipose tissue prior to injection. This system allowed the collection, microfragmentation, and filtration of tissue.

**Results:**

Selected cases included six men and three women with a median age of 42 (range 31–55) years. All patients had an extended disease course period, ranging from 3 to 13 (mean 6.6) years, and a history of multiple previous surgeries, including two to eight interventions (a mean of 4.4 per case). All fistulas were high transsphincteric, four cases horseshoe and two cases with secondary suprasphincteric or peri-elevator tract fistulas. Six cases (66%) achieved complete fistula healing at a mean follow-up of 18 (range 12–36) months. Three cases (33.3%) experienced reduced secretion and decreased anal discomfort.

**Conclusions:**

In patients with complex and recurrent fistulas, such as the ones described, many from palliative treatments with setons, the adjuvant injection of adipose tissue might help achieve complete healing or improvement in a significant percentage of cases.

**ClinicalTrials:**

The study protocol was prospectively registered on ClinicalTrials.gov (NCT 04750499).

## Introduction

Complex fistulas are those that have multiple external openings, involve > 30% of the external anal sphincter, are located above the sphincters (suprasphincteric), have large blind extensions or horseshoe-shaped trajectories, are in patients with pre-existing incontinence or Crohn’s disease (CD), or have an anterior location in women [1, 2].

Treatment of these complex anal fistulas is an unresolved issue. Surgery, the only curative treatment, obtains suboptimal results with high percentages of persistence or recurrence and a high risk of postoperative fecal incontinence [2, 3]. Recurrent anal fistulas after previous surgery are usually associated with a higher risk of re-recurrence and continence disturbance [2, 3]. In some cases, palliative treatment with long-term setons is considered [4, 5].

In this scenario, the association of surgery with stem cell transplantation has been considered, aiming to regenerate tissues and promote healing [6]. In fistulas related to CD, treatment with stem cells seems to offer hope, with remission rates of 51.5–56% in the treatment group vs. 35.6–40% in the control group [7–9]. In complex fistulas of cryptoglandular origin, stem cells in combination with simple surgery have also obtained promising results, with healing rates close to 60% [6, 10–12].

Autologous or allogeneic transplantation of non-commercial cultured stem cells is a complex procedure subjected to strict safety regulations and unavailable outside the scope of research projects. Using commercially available cultured allogeneic stem cells (Darvadstrocel-Alofisel®) is a highly expensive technique, costing between $65,000 and $87,000 per therapy [13, 14]. The Spanish public health system covers the cost of Alofisel® for CD fistulas but not for cryptoglandular fistulas.

An alternative to allogeneic stem cell treatment could be injecting freshly collected autologous adipose tissue [13, 15]. This treatment has been effective for CD-related and cryptoglandular fistulas [13–16]. Much evidence supports the regenerative capacity of adipose tissue and the association between these effects and the presence of stem cell precursors in these tissues [17]. These adipose tissue stem cells are known as adult mesenchymal stem cells (AMSCs), also called mesenchymal stromal cells.

This study aimed to investigate the efficacy of adipose tissue injection in treating patients with complex and recurrent fistulas of cryptoglandular origin. Injection of autologous adipose tissue, in addition to a surgical technique, combines the benefits of surgery with those of regenerative medicine, hoping that the beneficial effects of AMSCs will aid in the healing and repair process. In this preliminary study, we decided to limit the indications to cases of complex and recurrent fistulas.

## Methods

We conducted a prospective, non-randomized, interventional, single-center, open-label clinical trial from January 2020 to December 2022. The study enrolled nine patients and assessed them after a minimum of 12 months of follow-up. The study was approved by the Hospital Clinic Ethics Committee (reg. HCB/2020/0935) and was conducted following the Declaration of Helsinki. The study protocol was prospectively registered on Clinicaltrials.gov (NCT04750499).

**Inclusion criteria:** patients over 18 years of age, with complex and recurrent fistulas, diagnosis confirmed by magnetic resonance imaging (MRI) or endoanal ultrasound (EUAS), and written informed consent signed.

**Exclusion criteria:** Crohn’s disease, active septic process, pregnancy, patient unable to follow the pathway required by the protocol, and failure to sign the informed consent form.

### Surgical optimization before undergoing definitive surgery

We scheduled an optimization procedure for 4–6 weeks before definitive surgery in all cases. This procedure included exploration under anesthesia, exclusion of active infections or cavities, curettage of tracts or unroofing of cavities, and implantation of one or more loose setons (in most cases changing previous setons). In cases where the external fistula opening was far from the anus (long fistula tract), a modified rerouting procedure was also applied [18, 19]. In one case, the tracts were rerouted to the interesphinteric space; in two more cases, the tracts were rerouted only to approximate the external opening to the external anal sphincter and convert large fistulous tracts into short tracts to facilitate healing.

### Surgical procedure

The collection of autologous adipose tissue, fistula tract treatment, and injection of filtered adipose tissue were performed in a single surgical procedure under spinal anesthesia. A prophylactic intravenous antibiotic (2 g cefotaxime) was administered before surgery. We did not administer preoperative enema or mechanical bowel preparation.

The patient was supine for the liposuction step, which was always performed in the anterior abdominal wall. In six cases, we used the jackknife position for the fistula treatment step. In two cases with a high posterior internal fistula opening, we used the lithotomy position to make the advancement flap technique easier.

We collected and processed the adipose tissue using the Lipogems® system (Lipogems International SpA, Milan, Italy), which comes with a kit that contains all the necessary components for collection (liposuction needle and syringe), processing (filter and washing system), and injection (injection needles). Before harvesting the adipose tissue, we added a mixture of 10 ml 2% lidocaine (200 mg) and 0.5 mg adrenaline to 500 ml saline; 200–300 ml of this modified Klein solution was injected into the subcutaneous adipose tissue of the lower abdomen. After a gentle massage, liposuction was performed using a 13G blunt cannula with side holes connected to a VacLock 20-ml syringe. In each case, we collected approximately 100–150 ml adipose tissue and immediately processed it with the Lipogems® device as previously described [17, 20]. The mechanical processes of microfracturing, washing, and filtration take place in this system completely immersed in physiological solution, avoiding the presence of air, to make the reduction in volume possible and minimize any traumatic action on cellular products. This process reduces the size of the fragments of adipose tissue while eliminating oily substances and blood residues, which have pro-inflammatory properties. The resulting microfragmented and filtered adipose tissue (20–30 ml) was collected in 10-ml syringes. Finally, the product was transferred to several 2-ml syringes to be injected into the patient. A schematic representation of the successive steps in fat processing can be seen in the papers of Tremolada [17] and Bianchi [20].

If possible, a “core-out” fistulectomy, whether entire or partial, was performed. We performed vigorous curettage of the fistula tract using a metallic curette in situations with a weak external sphincter, followed by irrigation with saline solution. External openings were excised, and internal openings were sutured with 000 polyglactin absorbable sutures (Novosyn, B. Braun, Rubí, Barcelona, Spain). If local tissue conditions allowed, a mucosal flap was dissected and sutured with 000 absorbable sutures (Novosyn, B Braun, Rubi, Barcelona, Spain) over the internal opening suture. In all cases, adipose tissue was injected with a 22G and 30-mm-length needle in the mucosal and muscular layers around the internal opening (6 ml) and the mucosal flap (4 ml). Approximately 10–15 ml adipose tissue was injected along the fistula tract until firm swelling was obtained.

Patients were discharged after a clinical evaluation on the day of surgery. Postoperative treatment consisted of dexketoprofen (25 mg/8 h po) and paracetamol (1000 mg/8 h po). No postoperative antibiotics were administered. We advised the patients to take a mild laxative if they were constipated and to perform a sitz bath every 8 h and after every bowel movement.

At the end of the surgical procedure, we registered the characteristics of the fistula tract, intraoperative problems, and the volume of injected fat. At each postoperative follow-up visit, we prospectively evaluated complications such as fever, bleeding, abdominal or perineal hematoma, or abscesses.

The primary end point was fistula healing at clinical examination 12 months after the last injection. Complete fistula healing was defined as the closing of the internal and external openings without any discharge or symptoms. Secondary end points were (1) achieving reduced secretion and anal discomfort in patients who did not completely heal and (2) treatment-related complications. Follow-up visits were scheduled for 10 days and 1, 3, 6, and 12 months postoperatively. If the patient was asymptomatic, a clinical examination, including a digital rectal examination, was accepted to evaluate the outcome. An MRI was used to assess the situation in symptomatic patients with persistence or recurrence of discharge. Impaired continence was assessed before and after the surgery using the Wexner score [21].

## Results

**Fig. 1:**
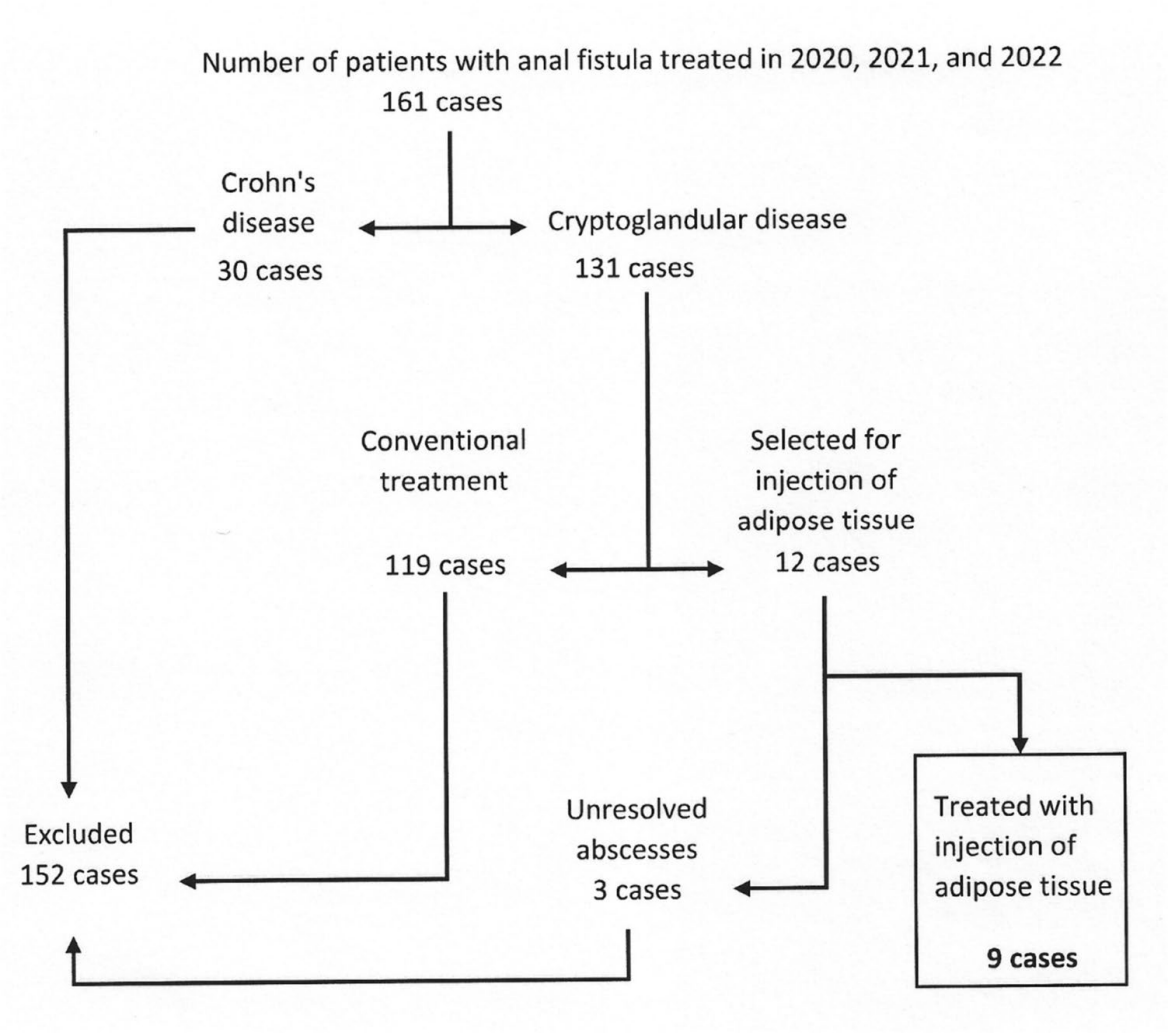
Case selection flow chart

Between January 2020 and December 2022 (see Fig. 1, flow chart), 131 cases of cryptoglandular anal fistulas were surgically treated in our hospital; 30 more cases of fistulas related to CD were operated on in a separate protocol that included seton drainage or treatment with allogeneic expanded adipose-derived stem cells (Darvadstrocel-Alofisel®). Twelve cryptoglandular cases (9.2%) were initially chosen for combined treatment with injection of adipose tissue according to selection criteria. Three patients were later excluded because they had unresolved abscesses discovered during the preliminary optimization procedures. We treated these cases with new deroofings, curettages, and seton maintenance; we will reevaluate them once the abscesses heal.

The final selected cases (Table 1) included six men and three women with a mean age of 41.6 (31–55) years. The nine included cases represented 6.9% of the cryptoglandular fistulas operated on at our center during the study period. Five cases corresponded to patients initially operated on in other centers. In five cases, a waiver of cure was originally assumed using palliative setons. All cases had an extended course period, ranging from 3 to 13 (average 6.6) years, and a history of many previous surgeries (two to eight interventions, with an average of 4.4 per case). EUAS and MRI investigations revealed high transsphincteric fistulas in all cases, horseshoes in four cases, and secondary suprasphincteric or perielevator tracts in two cases. In all cases, there was a non-severe alteration in continence before surgery, primarily in the form of “soiling;” preoperative Wexner scores are shown in Table 1.

**Table 1:** Characteristics of patients

Case number	1	2	3	4	5	6	7	8	9
Indications for study inclusion	High tract Recurrences	High tract Recurrences	High tract Recurrences 3 years on seton	High tract Recurrences Horse-shoe anterior in woman	High tract Recurrences Assumed palliative seton	High tract Recurrences Assumed palliative seton	High tract Recurrences/ Suggested palliative seton	High tract Recurrences Assumed palliative seton	High tract Recurrences Palliative seton. Anterior in woman
Age	38	55	31	33	52	35	48	48	34
Sex	Female	Male	Male	Female	Male	Male	Male	Male	Female
BMI	32	36	27	23	32	28	46 (34)	29	47
Comorbidities and other conditions	Obesity, Systemic lupus (SLE). Hidradenitis	Obesity Diabetes Pilonidal disease	HIV, Anal Lues	Severe ischiorectal abscess in pregnancy	Testicular cancer (disease free)	Hidradenitis Pilonidal disease	Obesity, Diabetes	No	Obesity
Years of evolution	3	3	3	3	9	13	3	11	11
Previous surgeries	3	2	2	3	6	7	3	8	6
Pre. Wexner	4	4	4	4	4	4	4	4	4
Diagnostic	EAUS, MRI	EAUS, MRI	EAUS, MRI	EAUS, MRI	EAUS, MRI	EAUS, MRI	EAUS, MRI	EAUS, MRI	EAUS, MRI
Parks classification	High perielevator Horseshoe	High transsphinteric vs supra. Horseshoe	High transsphinteric	High transsphinteric Horseshoe	High transsphinteric	High transsphinteric (including all EAE severely injured)	High transsphinteric	High transsphinteric	High transsphinteric Horseshoe
Number of the fistula tract	2 (posterior)	2 (posterior)	1 (anterior)	2 (Anterior)	4 (lateral and posterolateral) Long course	1 (posterior),	1 (very long lateral course)	2 (laterals) Long course	2 (anterior)
Length of fistula tract	3.4 cm	2.9 cm	2 cm	4 cm	6 cm	2 cm	6 cm	5 cm	2 cm

*HIV* human immunodeficiency virus, *BMI* body mass index, *SLE* systemic lupus erythematosus, *EAE* external anal sphincter, *EAUS* endoanal ultrasound, *MRI* magnetic resonance imaging

In two cases, a pilonidal disease in the sacrococcygeal region coexisted with anal fistula; in both cases, there was histological confirmation of pilonidal disease, and EAUS, MRI, and serum injection identified anal fistula existence. In one instance, the pilonidal disease was treated before derivation to our hospital, and in the other, a Limberg flap was performed 6 months after anal fistula treatment.

As shown in Table 2, each patient received a "tailored" treatment during definitive surgery, with fat injection working as the unifying characteristic. All patients underwent removal of the seton, fistula “core-out” or curettage, transanal closure of the internal opening by suture or a mucosal flap (performed in six cases), and adipose tissue injection. In one case with suprasphincteric fistula, the internal opening suture could not be carried out; therefore, extensive curettage and fat injection were used. The volumes of adipose tissue finally injected ranged between 10 and 30 (mean 20.4) ml. Two to 3 months after the initial procedure, two patients underwent a second procedure that included new curettage and adipose tissue reinjection.

**Table 2:** Surgical details and outcomes

Case number	1	2	3	4	5	6	7	8	9
Surgical optimization	Setons and drainages	Setons and drainages	Curettage and seton	Tracts rerouting to intersphinteric space + 2 setons	Tracts rerouting to approximate the external opening. Curettage + 2 setons	Curettage and seton	Tract rerouting to approximate the external opening. Curettage and seton	Cavity unroofing, curettage, and 2 setons	Curettage and 2 setons
Definitive treatment of tract	Fistulotomy low tract + curettage perielevator tract	Tract core-out + high tract curettage	Tract core-out	Partial fistulotomy rerouted tracts	Partial core-out + curettage	Curettage	Almost complete core out resection	Curettage	Curettage
Closure of IO	No closure	IO suture + mucosal flap	IO suture + mucosal flap	IO suture + Mucosal flap	IO suture + mucosal flap	IO suture	IO suture + mucosal flap	IO suture + mucosal flap	IO suture
Injected volume	20 ml	20 ml	18 ml	10 ml	20 ml	23 ml	30 ml	25 ml	18 ml
Time closure	3 mos	12 mos	3 mos	2 mos	No	No	2 mos	2 mos	No
Complications of procedures	No	No	No	Hemorrhage in the skin margin	No	No	No	Pain 1 month	No
Post-op. Wexner	0	8	0	0	4	4	0	0	4
Further surgery during follow-up	No	(1) Limberg flap for pilonidal disease. (2) Tract curettage + adipose tissue reinjection	No	No	(1) Debridement and curettage residual external opening	(1) Debridement and curettage residual EO (pilonidal disease?)	No	No	(1) Reoperation with IO suture + adipose tissue reinjection
Follow-up	34 mos	38 mos	25 mos	21 mos	23 mos	20 mos	12 mos	14 mos	12 mos
Final result	Complete healing	Complete healing	Complete healing	Complete healing	Improvement. Persistent low suppuration in EO	Improvement. Minor abscess without IO communication	Complete healing	Complete healing	Improvement. 1 tract closed; 1 tract open. Low suppuration
Result evaluation	Clinical	Clinical	Clinical	Clinical	Clinical and MRI	Clinical and MRI	Clinical	Clinical	Clinical

*MRI* Magnetic resonance imaging; *IO* Internal opening; *EO* External opening; *ml* Milliliters; *mos* Months

Six cases (66.6%) showed complete healing at a mean follow-up of 22 (range: 12–38) months, and three cases (33.3%) showed a significant reduction in secretion and discomfort. Five cases achieved complete healing with a single operation. In another case, a new curettage of a residual cavity combined with a new fat injection resulted in complete healing of the fistula tract.

The mean time to achieve total or partial healing was 2 months. There were no complications derived from liposuction or adipose tissue injection. One patient presented a minor hemorrhagic complication after preparatory curettage of the fistula tracts. Only one patient reported deterioration in fecal continence. In five cases, continence improved with the disappearance of soiling; postoperative Wexner scores are shown in Table 2.

MRI: magnetic resonance imaging; IO: internal opening; EO: external opening; ml milliliters; mos: months.

## Discussion

We report the successful use of a combined sphincter-sparing surgical technique with adjuvant fat tissue injection in patients with complex and recurrent cryptoglandular anal fistulas. We aimed to enhance the healing power of the surgery and decrease the risk of incontinence by adding the regenerative effect of adipose tissue injection in these apparently untreatable cases.

In this preliminary study, we decided to limit the indications of adipose tissue injection to cases of extremely complex and recurrent fistulas, many under palliative treatments with setons. The included cases represented 6.9% of the cryptoglandular fistulas operated on at our center during the study period. We believe a basic fistulotomy can heal > 70% of fistulas [2], and other standard procedures like LIFT or “core-out” fistulectomy with mucosal flap can treat a substantial proportion of the remaining, more complex cases.

In our experience, microfragmented adipose tissue injection resulted in full healing in six of nine cases (66%) of complex and recurrent fistulas. The remaining three patients significantly improved compared with their previous situation. We believe that our results in these extremely complex circumstances are quite satisfactory.

In our proposed approach, preparing the patient beforehand with the unroofing and curettage of intermediate cavities is very important. We have excluded cases with untreated intermediate cavities and will reconsider them once they resolve. Rerouting techniques [18, 19] to convert large fistulous tracts, with an external opening far from the anus, into short tracts can facilitate healing.

The standardization of surgical treatment of the fistula tract in such severe cases is very difficult. We prefer a "core-out" excision, whether entire or partial. We perform a partial core-out with curettage of the intrasphincteric portion when excising the tract inside the sphincter is difficult or dangerous. We also believe that, whenever possible, treating the internal opening with a mucosal flap improves the outcomes.

Previous studies have shown that injecting adipose tissue is a therapeutic alternative for treating cryptoglandular fistulas [13, 22, 23]. In the only existing randomized study, Ascanelli [23], associating various surgical techniques with fat injection in 58 patients per group, found no differences in the percentage of healing at 6 months (86.2% vs. 81.3% in the control group). However, improvement in the mean healing time was very significant (16 vs. 60 days). In a recent study, Dalby [13] found that adipose tissue injection along with simple suturing of the internal fistula opening led to 51% clinical healing and a 12% decrease in secretion and pain. Naldini [22] published 19 cases of patients with complex fistulas unrelated to CD: 12 primary and 7 relapsed. Treatment consisted of the closure of the internal opening and the injection of adipose tissue. The healing rate was 83.3% in primary fistulas and 57.1% in previously relapsed fistulas. Another series [24] treated ten cases of complex fistulas unrelated to CD with simple internal opening sutures and adipose tissue injection, achieving a 70% healing rate at 9 months. In another small series of nine patients with five cases of multiple relapses, Tutino [25] proposes a combination of adipose tissue and platelet-rich plasma (PRP) injection, showing a 66% healing rate at 2 years.

Adipose tissue injection is a low-cost and technically simple procedure, although the heterogeneity of lipoaspirate preparation methods (centrifugation, filtration, microfragmentation, among others) makes standardization of the technique difficult [26]. In our study, we used the Lipogems® system [17], a sterile, single-use closed circuit, that avoids the risks of using centrifuges and non-specific filters. In addition, there are numerous studies that describe its final product and the types of cells it contains [17, 20, 27–29].

Injection of adipose tissue has become increasingly popular in other fields, showing promising results in treating diabetic foot ulcers and other chronic scars [30–32]. The effects of adipose tissue injection on tissue regeneration and the healing of chronic wounds are poorly understood. Proangiogenic, antiapoptotic, antifibrotic, immunoregulatory, anti-inflammatory, and trophic properties of the stromal vascular fraction of adipose tissue are widely recognized [27, 30, 32, 33]. Some authors suggest AMSCs are able to differentiate and grow in their transplanted environment, regenerating surrounding tissues [34]. Furthermore, the paracrine effects of AMSCs may result from the secretion of cytokines, such as different growth factors or granulocyte-macrophage colony-stimulating factors [34]. Other biological treatments, like the application of platelet-rich plasma (PRP) injection in anal fistulas, argue for similar effects. Biologically active factors released by platelets, such as cytokines, growth factors, adhesion proteins, and others, can initiate tissue repair and induce revascularization [35, 36].

Another aspect to analyze is the possible beneficial effect of adipose tissue injection on the preservation of continence. Evidence shows that even sphincter-sparing surgical techniques cause high incontinence rates [2]. Injection of adipose tissue into sphincters has been described as a treatment for fecal incontinence [37–40]. In this regard, we suggest that the injection of adipose tissue may have significantly influenced the preservation or improvement of our patients’ continence.

There is no doubt that the current study has substantial limitations. This initial experience only included a few patients, with no control group. Other drawbacks include the variability of clinical situations and the individually tailored surgical techniques used for each patient. Additionally, it is difficult to discern between the injected adipose tissue and the advancing flap’s contributions to healing. We are on a learning curve regarding patient selection, preparation, and the best surgical method to use in conjunction with cellular injection. Additional work is required to develop appropriate treatment protocols for these challenging cases.

## Conclusions

In patients with complex recurrent fistulas, such as the ones described, many from palliative treatments with setons, the adjuvant injection of adipose tissue might help achieve complete healing or improvement in a significant percentage of cases.

## Data Availability

The regulations stated in our institutional policies prevent us from making the dataset we generated and analyzed for this study publicly available. The first author could provide de-identified data upon reasonable request.

## Abbreviations

CD: Crohn disease
AMSCs: Adult mesenchymal stem cells
EAUS: Endoanal ultrasound
MRI: Magnetic resonance imaging
